# Acetylated Resveratrol and Oxyresveratrol Suppress UVB-Induced MMP-1 Expression in Human Dermal Fibroblasts

**DOI:** 10.3390/antiox10081252

**Published:** 2021-08-05

**Authors:** Jae-Eun Lee, Jijeong Oh, Daeun Song, Mijeong Lee, Dongyup Hahn, Yong Chool Boo, Nam Joo Kang

**Affiliations:** 1 School of Food Science and Biotechnology, Kyungpook National University, Daegu 41566, Korea; lju1033@naver.com (J.-E.L.); ojjeong0113@hanmail.net (J.O.); sde940902@naver.com (D.S.); lmj7083@hanmail.net (M.L.); dohahn@knu.ac.kr (D.H.); 2 Department of Integrative Biology, Kyungpook National University, Daegu 41566, Korea; 3 Department of Molecular Medicine, Cell and Matrix Research Institute, BK21 Plus KNU Biomedical Convergence Program, School of Medicine, Kyungpook National University, Daegu 41944, Korea; ycboo@knu.ac.kr

**Keywords:** resveratrol, oxyresveratrol, acetylated derivative, matrix metalloproteinase-1, type I collagen, ultraviolet B, skin aging

## Abstract

Resveratrol (RES) and oxyresveratrol (OXYRES) are considered and utilized as active ingredients of anti-aging skin cosmetics. However, these compounds are susceptible to oxidative discoloration and unpleasant odor in solutions, limiting their use in cosmetics. Accordingly, RES and OXYRES were chemically modified to acetylated derivatives with enhanced stability, and their anti-aging effect on the skin and detailed molecular mechanism of their acetylated derivatives were investigated. Acetylated RES and OXYRES lost their acetyl group and exerted an inhibitory effect on H_2_O_2_-induced ROS levels in human dermal fibroblast (HDF) cells. In addition, RES, OXYRES, and their acetylated derivatives suppressed UVB-induced matrix metalloproteinase (MMP)-1 expression via inhibition of mitogen-activated protein kinases (MAPKs) and Akt/mTOR signaling pathways. Furthermore, RES, OXYRES, and their acetylated derivatives suppressed type I collagen in TPA-treated HDF cells. Collectively, these results suggest the beneficial effects and underlying molecular mechanisms of RES, OXYRES, and their acetylated derivatives for anti- skin aging applications.

## 1. Introduction

Skin aging is a biological phenomenon involving intrinsic and extrinsic pathways and is phenotypically characterized by skin roughness, pigmentation, and decreased elasticity. Intrinsic aging is a process that occurs over time, whereas extrinsic aging is caused by exposure to environmental factors [1]. Ultraviolet (UV) radiation exposure is the most important environmental factor associated with premature facial aging (photoaging) [2].

UV light can be divided into three types depending on the wavelength: UVA (320–400 nm), UVB (280–320 nm), and UVC (200–280 nm). UVB is the most active constituent of solar light. It provokes the generation of reactive oxygen species (ROS), impairs the skin’s antioxidant status, and causes damage to DNA and proteins. Both direct and indirect adverse biological effects induced by UVB can lead to photocarcinogenesis and photoaging, which is clinically characterized as wrinkle formation and loss of skin elasticity [3]. UVB-induced degradation of the extracellular matrix (ECM), as well as decreased expression of collagen and increased expression of matrix metalloproteases (MMPs), can also contribute to photoaging [4,5].

MMPs are secreted from fibroblasts and keratinocytes and degrade the collagen and other proteins in the ECM [6]. Among them, MMP-1 (collagenase) plays a major role in the specific degradation of collagen types I and III during the aging process of the human dermis [7], suggesting that inhibition of MMP-1 induction could reduce UV-induced photoaging [8,9,10]. However, UV irradiation also activates complex signal transduction pathways mediated by growth factor receptors and members of the mitogen-activated protein kinase (MAPK) family, such as extracellular signal-regulated kinases (ERKs), c-Jun N-terminal kinases (JNKs), and p38 via ROS [11]. The transcription factor activator protein-1 (AP-1) is a major effector of the MAPK pathway and regulates the transcription of several MMP family members that together are capable of degrading all the ECM proteins [11,12].

Resveratrol (3,4′,5-trihydroxy-trans-stilbene; RES) is a natural stilbenoid polyphenol with three hydroxyl groups, which has been demonstrated to have cosmeceutical properties as well as anti-proliferative, antioxidant, anti-inflammatory, anti-angiogenic, and anti-metastatic effects in many different cell lines [13,14,15]. Oxyresveratrol (2,3′,4,5′-tetrahydroxy-trans-stilbene; OXYRES) is a stilbenoid polyphenol derivative with one more hydroxyl group of RES found in some plants and has a potent tyrosinase-inhibiting activity, among other properties [16]. However, RES and OXYRES are photosensitive and display poor solubility, rendering them susceptible to oxidative discoloration and unpleasant odor in solution. These problems impede their industrial utilization in cosmetics, especially when exposed to light and heat [17]. There have been various approaches to overcome these limitations [17,18]. In our previous study to improve the application of RES and OXYRES, the compounds were chemically modified to acetylated derivatives [19]. Compared with the parent compounds, triacetyl RES (AcRES) and tetraacetyl OXYRES (AcOXYRES) demonstrated chemical stability against heat and light, allowing long-term storage. In addition, the previous study showed that acetylated RES and OXYRES could be used as skin whitening agents in B16F10 melanocytes.

We hypothesized that the as-prepared and chemically stable AcRES and AcOXYRES would be reduced back to their parent compounds in human dermal fibroblast (HDF) cells and consequently also exert beneficial anti-aging effects on skin based on the antioxidant activity of their parent compounds. Therefore, in this study we examined the protective effects and molecular mechanisms of acetylated RES and OXYRES on UVB-induced skin aging to determine their utilization as cosmeceuticals.

## 2. Materials and Methods

### 2.1. Chemicals and Antibodies

**Figure 1:**
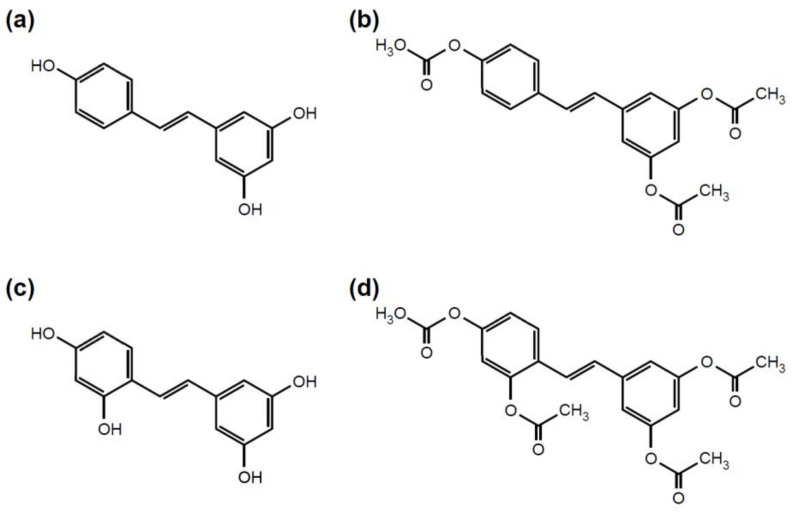
Chemical structures of (**a**) resveratrol (RES), (**b**) triacetyl resveratrol (AcRES), (**c**) oxyresveratrol (OXYRES), and (**d**) tetraacetyl oxyresveratrol (AcOXYRES).

RES, AcRES, OXYRES, and AcOXYRES (Figure 1) were provided by Dr. Yong Chool Boo of Kyungpook National University, Daegu, Korea [19]. L-Ascorbic acid, 3-(4,5-dimethylthiazol-2-yl)-2,5-diphenyltetrazolium bromide (MTT), 2,2-diphenyl-1-picryl-hydrazyl (DPPH), 2,2′-azino-bis(3-ethylbenzothiazoline-6-sulfonic acid) diammonium salt (ABTS), porcine liver esterase, 4-nitrophenyl acetate, and dichloromethane (CH_2_Cl_2_) were purchased from Sigma Chemical Co. (St. Louis, MO, USA). The MMP-1 Fluorometric Drug Discovery Kit and TQ3-GABA-Pro-Cha-Abu-Smc-His-Ala-Dab(6-TAMRA)-Ala-Lys-NH_2_ [TQ3 = quencher; GABA = 4-aminobutyric acid; Cha = L-cyclohexylalanine; Abu = 2-aminobutyric acid; Smc = S-methyl-L-cysteine; Dab = 2,4-diaminobutyric acid; 6-TAMRA = 6-tetramethylrhodamine] were obtained from Enzo Life Sciences, Inc. (Farmingdale, NY, USA). Anti-human MMP-1 antibody (Neomarkers, Inc., Fremont, CA, USA) and anti-β-actin antibody (Sigma-Aldrich, St. Louis, MO, USA) were purchased from the indicated commercial suppliers. The antibodies against phosphorylated Raf-1, total Raf-1, phosphorylated MEK1/2 (Ser217/221), total MEK1/2, phosphorylated MKK4/7, total MKK4/7, phosphorylated MKK3/6, total MKK3/6, phosphorylated p90RSK, total p90RSK, phosphorylated p38, phosphorylated JNK1/2, total JNK1/2, phosphorylated Akt, total Akt, phosphorylated mTOR, total mTOR, phosphorylated p70S6K, and total p70S6K were purchased from Cell Signaling Biotechnology (Danvers, MA, USA). The antibodies against phosphorylated ERK1/2, total ERK1/2, and total p38 were procured from Santa Cruz Biotechnology (Santa Cruz, CA, USA).

### 2.2. Cell Lines and Medium

Normal HDF cells were provided by Dr. Jin Ho Chung (College of Medicine, Seoul National University, Seoul, Korea). Dulbecco’s modified Eagle’s medium (DMEM), penicillin–streptomycin, 0.5% trypsin–EDTA, and fetal bovine serum (FBS) were purchased from Gibco (Grand Island, NY, USA).

### 2.3. Cell Culture

HDF cells were cultured in DMEM supplemented with 10% FBS and 1% penicillin–streptomycin by incubation at 37 °C in a humidified atmosphere containing 5% CO_2_. Cultured HDF cells at passages 10–18 were used for experiments.

### 2.4. MTT Assay

HDF cells were seeded (3 × 10^3^ cells/well) in 96-well plates and incubated at 37 °C for 24 h to allow the cells to attach. The attached HDF cells were treated with each compound at various concentrations. Afterward, 20 μL of MTT solution (final concentration, 1 mg/mL) was added to each well, and the cells were incubated for 2 h. The cell culture medium was subsequently removed, and each well was treated with 200 μL DMSO to dissolve formazan crystals. Dissolved formazan absorbance was measured at 570 nm using a microplate reader (Sunrise-Basic Tecan, Tecan Austria GmbH, Grödig, Austria). The cell viability was expressed as the percentage of MTT reduction calculated relative to the absorbance of control cells. In addition, the concentration that reduced the cell viability to 50% was calculated as the cytotoxic concentration 50 (CC50).

### 2.5. Antioxidant Activity

#### 2.5.1. DPPH Assay

The method of Brand-Williams et al. was used with slight modifications [20]. DPPH radical solution was dissolved in 80% (*v/v*) aqueous methanol to a final concentration of 0.4 mg/mL. Then, 10 μL of the compounds at the appropriate concentration and 190 μL of the diluted DPPH radical solution were mixed in a 96-well plate. The mixture was shaken vigorously and allowed to stand at room temperature in the dark for 30 min, at which time the decrease in absorbance at 517 nm was measured by a microplate reader (Sunrise-Basic Tecan, Tecan Austria GmbH).

#### 2.5.2. ABTS Assay

ABTS was dissolved in methanol to a final concentration of 7 mM. To generate ABTS^+•^ the methanolic ABTS solution was reacted with 2.45 mM potassium persulfate (final concentration) in the dark at room temperature for 12–16 h until the reaction was complete and the absorbance was stable. For analysis of the ABTS radical scavenging activity, the ABTS^+•^ stock solution was diluted in ethanol to an absorbance of 0.7 ± 0.02 at 734 nm, then 90 μL of the diluted ABTS^+•^ solution was added to 10 μL of the sample and mixed. Five minutes later, the absorbance was read at 734 nm [21].

#### 2.5.3. H_2_DCFDA (2′,7′-dichlorofluorescin diacetate) Assay

HDF cells were cultured in a black 96-well plate at 4 × 10^3^ cells/well and incubated for 24 h. Cultured HDF cells were further incubated with 20 μM of indicated compounds for 12 h, followed by evaluation of H_2_O_2_-induced ROS generation levels. The cells were washed twice with Hank’s Balanced Salt Solution (HBSS) and treated with 20 μM of H_2_DCFDA in the dark. After 1 h, H_2_DCFDA was removed, and the cells were washed twice with HBSS. Subsequently, 0 or 30 μM H_2_O_2_ was treated for 0.5 h to generate intracellular ROS. The ROS levels were measured by detecting the fluorescence signal at λ_e*xcitation*_/λ_e*mission*_ (λ*_ex_*/λ*_em_*) = 485/535 nm and calculated as the fold change relative to the untreated control.

### 2.6. Deacetylation of AcRES and AcOXYRES

#### 2.6.1. Hydrolysis of the Acetylated Compounds

HDF cells were harvested at about 90% confluence using cell lysis buffer (20 mM Tris–HCl (pH 7.5), 150 mM NaCl, 1 mM Na_2_EDTA, 1 mM EGTA, 1 mM Na_3_VO_4_, 1 mM PMSF, 1 mM glycerophosphate, 1% Triton X-100, 2.5 mM sodium pyrophosphate, 1 μg/mL leupeptin, and a protease inhibitor cocktail tablet). After splitting the cell lysate in half, one half was kept fresh, and the other half was heat-treated at 90 °C for 20 min to inactivate the enzyme. The protein concentration of each cell lysate was quantified using a Bio-Rad Protein Assay Kit (Bio-Rad Laboratories, Hercules, CA, USA), as described in the manufacturer’s manual. Fresh or heat-treated HDF cell lysates containing 80 μg protein or cell lysis buffer were mixed with 100 μM RES, AcRES, OXYRES, or AcOXYRES in 100 mM sodium phosphate buffer (pH 6.8). Each reaction mixture was prepared with a final volume of 200 μL and incubated at 37 °C for 24 h. Additionally, 100 μM RES, AcRES, OXYRES, or AcOXYRES were incubated with esterase (0, 2, or 10 U/mL) in 100 mM sodium phosphate buffer (pH 6.8) at 37 °C for 24 h.

#### 2.6.2. Sample Preparation and HPLC Analysis

Each reaction mixture of acetylated RES or OXYRES with HDF cell lysates, heat-treated cell lysates, or only cell lysis buffer was extracted with 200 μL of ethyl acetate. The mixture was vortexed, and the organic layer was taken for analysis. Ethyl acetate was removed under reduced pressure, and the residue was dissolved in 200 μL of HPLC-grade acetonitrile and filtered through a syringe filter. Standard RES, OXYRES, AcRES, and AcOXYRES were dissolved in HPLC-grade acetonitrile at a concentration of 0.01 mg/mL. The reaction mixture of acetylated RES or OXYRES with esterase (0, 2, or 10 U/mL) was also prepared with the same procedure for HPLC analysis.

RES, OXYRES, and their acetylated derivatives were analyzed using an Alliance 2695 HPLC system (Waters, Milford, MA, USA), equipped with a diode array UV–visible detector (Waters 2996, Waters), and a Hector-M C18 column (250 × 4.6 mm, 5 μm; RStech, Daejeon, Korea). The mobile phases used were 0.1% trifluoroacetic acid in water (A) and acetonitrile (B) at a flow rate of 1.0 mL/min. The gradient elution program was set as follows: 0–5 min, 5% B; 5–20 min, 5–100% B; 20–25 min, 100% B; 25–30 min, 5% B. The column temperature was maintained at 40 °C throughout the analysis. The chromatograms were monitored at a wavelength of 280 nm, and the injection volume was 10 μL.

#### 2.6.3. Esterase Activity of HDF Cells

The esterase activity of HDF cells was measured using 4-nitrophenyl acetate, a substrate for esterase [22]. 4-Nitrophenyl acetate stock solution at 250 mM in CH_2_Cl_2_ was diluted to 0.5 mM using a 20 mM Tris–HCl (pH 8.0) buffer with 150 mM NaCl and 0.01% Triton X-100, just before the start of the analysis. HDF cells were harvested at about 90% confluence using cell lysis buffer (see Section 2.6.1). To confirm the esterase activity according to the concentration of HDF cell lysate, 20 μL of cell lysate at various concentrations was incubated with 200 μL of 0.5 mM working solution of the substrate at 37 °C for 10 min, and the absorbance was measured at 415 nm. In addition, fresh or heat-treated (90 °C, 20 min) HDF cell lysate containing 20 μg of protein was mixed with 0.5 mM 4-nitrophenyl acetate. Cell lysis buffer mixed with 0.5 mM 4-nitrophenyl acetate was prepared as the blank. The reaction mixture was incubated at 37 °C while absorbance was measured at 415 nm at 20-min intervals.

### 2.7. MMP-1 Activity

#### 2.7.1. In Vitro MMP-1 Activity Assay

The inhibitory effects of RES, OXYRES, AcRES, and AcOXYRES on MMP-1 enzymatic activity in a cell-free system were assessed using the MMP-1 Fluorometric Drug Discovery Kit (Enzo Life Sciences, Inc.) according to the manufacturer’s instructions. The assay kit included the specific MMP-1 inhibitor (NNGH), which served as the control inhibitor. The reaction mixtures were incubated at 37 °C for 5 min. The MMP-1 activities were measured by detecting the fluorescence signal at λ_e*x*_/λ_e*m*_ = 540/590 nm and calculated as the percentage relative to the enzyme-treated control.

#### 2.7.2. Intercellular MMP-1 Activity

The inhibitory effects of RES, OXYRES, AcRES, and AcOXYRES on intracellular MMP-1 enzymatic activity were determined by slightly modifying the in vitro MMP-1 activity assay (Section 2.7.1). HDF cells were harvested at about 90% confluence using M-PER Mammalian Protein Extraction Reagent (Thermo Fisher Scientific, Inc., Waltham, MA, USA) and quantified using a Bio-Rad protein assay kit (Bio-Rad Laboratories). HDF cell lysate containing 5 μg of protein and test compounds of 0, 5, 10, or 20 μM concentration were incubated at 37 °C for 30 min. Then, TQ3-GABA-Pro-Cha-Abu-Smc-His-Ala-Dab(6-TAMRA)-AlaLys-NH_2_ was added at a final concentration of 0.5 μM, and the fluorescence signal was detected at λ_e*x*_/λ_e*m*_ = 540/590 nm.

### 2.8. UVB Irradiation

HDF cells were starved for 12 h in serum-free DMEM medium and pretreated with indicated compounds for 1 h. Afterward, the cells were rinsed twice with warmed phosphate-buffered saline (PBS) and irradiated with 20 mJ/cm^2^ of UVB in a small volume of PBS. UVB irradiation was performed using a Bio-Link Crosslinker (Vilber Lourmat, Collégien, France) emitting wavelengths with peak emission at 312 nm. Immediately after UVB irradiation, pretreated media with indicated compounds were added to the HDF cells, which were cultured according to protein expression conditions (detailed in each figure caption).

### 2.9. Western Blot Analysis

HDF cells (6–8 × 10^4^) were cultured in a 60-mm diameter dish for 24 h, and then the cells were starved in serum-free DMEM for a further 12 h to eliminate the influence of FBS on the kinase activation. The cells were obtained using cell lysis buffer (see Section 2.6.1) after appropriate treatments, depending on the purpose of each experiment. The protein concentration of the cell lysate was determined (Section 2.6.1), and the proteins were separated by 8–10% sodium dodecyl sulfate–polyacrylamide gel electrophoresis and electrophoretically transferred to a polyvinylidene fluoride membrane (Millipore Corp., Bedford, MA, USA). The membrane was blocked in 5% skim milk and incubated with primary antibodies at 4 °C overnight, followed by incubation with secondary antibodies (HRP-conjugated anti-IgG). The antibody-bound proteins were visualized using an ECL Plus Western blotting detection system (GE Healthcare Life Sciences, Waukesha, WI, USA). β-Actin or the total formed kinase was used as the loading control. The protein expression level was calculated as the relative fold change to the UVB treatment group by measuring the band intensity using ImageJ from NIH (Bethesda, MD, USA).

### 2.10. Collagen Degradation Assay

The cell-mediated collagen assay was performed in 6-well cell culture plates coated with 1.5 mL of rat-tail tendon (RTT, 300 mg/mL) type I collagen per well, as previously described [23]. Briefly, RTT type I collagen was dissolved in a mixture of 13 mM HCl and neutralizing phosphate buffer (80 mL of 0.2 M NaH_2_PO_4_/Na_2_HPO_4_ buffer, 16.6 mL of 5 M NaCl, and 80 mL of 0.1 N NaOH) rapidly on ice to provide a final collagen concentration of 300 mg/mL and pH of 7.4. A 1.5-mL aliquot of neutralized collagen solution was dispensed in each well of the 6-well plates (0.45 mg of collagen gel) and incubated at 37 °C for 2–4 h to allow collagen gel formation. The collagen gels were dehydrated overnight under laminar air-flow in a hood, washed three times with distilled water, each time for 30 min, and then air-dried overnight in a laminar flow hood. The HDF cells (3×10^4^ cells in a total volume of 100 μL) were seeded in the center of each well. After allowing the cells to attach for 6 h, 2 mL of serum-free DMEM was added to each well with or without 150 nM 12-*O*-tetradecanoylphorbol-13-acetate (TPA; Sigma-Aldrich) and 20 μM of indicated compounds. After incubation for 5 days, the conditioned media was removed, the cells were removed with 0.25% trypsin, and then the wells were rinsed with distilled water. The washed wells were stained with Coomassie blue for 15 min to visualize residual collagen film and then destained with distilled water. Collagen degradation was analyzed using ImageJ from NIH.

### 2.11. Statistical Analysis

Quantitative data were presented as mean ± standard deviation (SD) and analyzed by one-way analysis of variance (ANOVA) using IBM SPSS Statistics 25 (IBM, Armonk, NY, USA). Statistical significance was identified by Duncan’s multiple range test at *p* < 0.05. Non-quantitative data represented three independent experiments that gave similar results.

## 3. Results and Discussion

### 3.1. AcRES and AcOXYRES Do Not Eliminate In Vitro Free Radicals but Reduce H_2_O_2_-Induced ROS in HDF Cells

RES is a potent antioxidant. OXYRES is similar in structure to RES and displays antioxidant activity [24,25]. Accumulation of ROS-induced oxidative damage, along with DNA damage and inflammation, are closely involved in skin aging [26]. Therefore, RES and OXYRES have been highly regarded as potential anti-aging agents for the skin. However, their chemical instability limits their industrial utilization in cosmetics. To this end, in a previous study, RES and OXYRES were reacted with acetic anhydride in an aprotic solvent to replace the hydroxyl groups of each compound with acetyl groups, resulting in the acetyl derivatives AcRES and AcOXYRES, respectively [19]. When these acetyl derivatives and their parent compounds were stored at a high temperature (60 °C) for 14 days, the RES and OXYRES solutions showed obvious discoloration, but not the AcRES and AcOXYRES solutions [19]. These results suggested that acetylation of RES and OXYRES could improve their oxidative stability.

**Figure 2:**
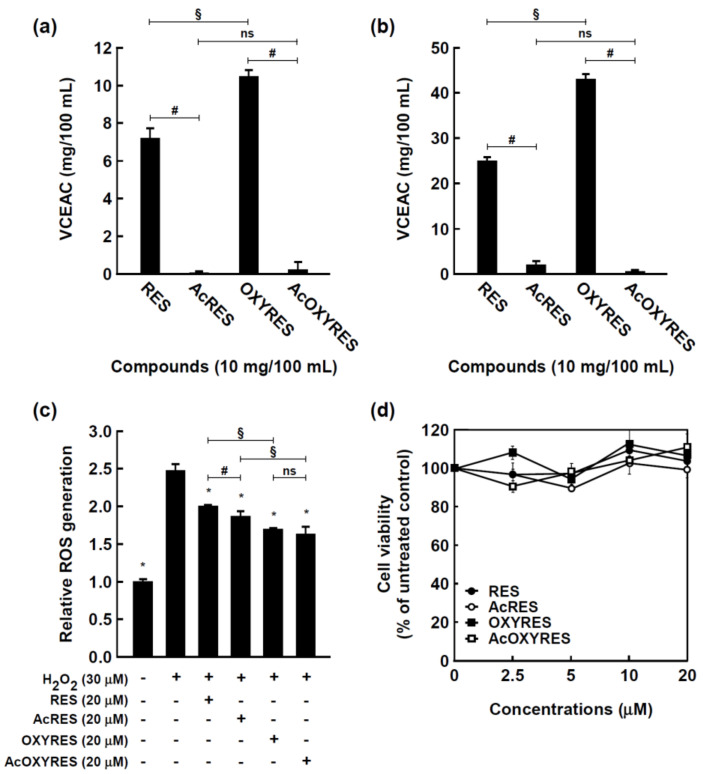
Antioxidative effects of RES and its derivatives. (**a**) 2,2-Diphenyl-1-picryl-hydrazyl (DPPH) radical scavenging activities and (**b**) 2,2’-azino-bis(3-ethylbenzothiazoline-6-sulfonic acid) diammonium salt (ABTS) radical scavenging activities of the compounds. Data represent mean ± standard deviation (SD) as vitamin C equivalent antioxidant capacity (VCEAC). (**c**) Intracellular reactive oxygen species (ROS) scavenging activities of the compounds in human dermal fibroblast (HDF) cells. The cells were incubated with or without RES and its derivatives for 12 h. Data are mean ± SD relative to the untreated control. (**d**) Cytotoxicity of RES and its derivatives on HDF cells. Cell viability was measured by the MTT assay as described in Section 2.4. *, Significant difference (*p* < 0.05) compared with H_2_O_2_-treated control; #, significant difference (*p* < 0.05) of parent vs. acetyl compounds; §, significant difference (*p* < 0.05) of (Ac)RES vs. (Ac)OXYRES; ns, not significant (*p* > 0.05).

To confirm whether AcRES and AcOXYRES could replace RES and OXYRES as anti-aging agents, we evaluated and compared the antioxidant activities of RES, OXYRES, AcRES, and AcOXYRES. In vitro DPPH^•^ scavenging activities of 10 mg/100 mL of RES and OXYRES were similar to vitamin C, with vitamin C equivalent antioxidant capacities of 7.2 ± 0.5 and 10.5 ± 0.3 mg/100 mL, respectively (Figure 2a). OXYRES, with one more hydroxyl group, had a significantly higher radical scavenging ability than RES. However, the results for AcRES and AcOXYRES, the acetylated derivatives of the parent compounds, were 0.0 ± 0.1 and 0.2 ± 0.4 mg/100 mL, respectively, indicating no DPPH^•^ radical scavenging activity (Figure 2a). The ABTS^+•^ scavenging capacities of RES, AcRES, OXYRES, and AcOXYRES at 10 mg/100 mL were equivalent to 24.9 ± 0.8, 1.9 ± 0.8, 42.9 ± 1.1, and 0.4 ± 0.3 mg/100 mL of vitamin C, respectively (Figure 2b). Like the DPPH results, the effect was higher for OXYRES than RES, and the acetylated derivatives had no ABTS^+•^ scavenging activity.

To further assess the antioxidant activities of RES, OXYRES, and their acetylated derivatives in HDF cells, the H_2_O_2_-induced intracellular ROS levels were determined by using the H_2_DCFDA assay. In contrast to the in vitro free radical scavenging effects, all compounds reduced H_2_O_2_-induced ROS levels significantly in HDF cells treated for 12 h, with similar or slightly better effects than the respective parent compounds. The intracellular ROS inhibition by AcRES was significantly higher than that of its parent compound RES, but it was not a dramatic difference, and there was no significant difference between OXYRES and AcOXYRES (Figure 2c). RES, AcRES, OXYRES, and AcOXYRES were treated at concentrations that did not have cytotoxicity in HDF cells for 24 h (Figure 2d). To additionally establish the CC50 value, the HDF cells were treated with comparatively much higher concentrations of RES, AcRES, OXYRES, and AcOXYRES for 24 h, and the results were 300.5 ± 55.6, 183.0 ± 22.4, 381.0 ± 37.4, and 182.6 ± 59.8 μM, respectively (Table S1).

These data indicated that OXYRES, with one more hydroxyl group, displayed higher free radical scavenging activity than RES; however, the acetylated forms, in which hydroxyl groups are replaced by acetyl groups, did not scavenge free radicals. These results are consistent with previous reports that the number of hydroxyl groups is related to the antioxidant activity of phenolic compounds [27,28]. However, we also observed that the intracellular ROS scavenging abilities in H_2_O_2_-treated HDF cells were maintained with acetylation of RES and OXYRES. Taken together, our findings suggested that acetylated RES and OXYRES cannot eliminate free radicals because of their molecular structure but that they can inhibit ROS generation in cells by inducing either chemical changes or other mechanisms.

### 3.2. AcRES and AcOXYRES Are Hydrolyzed to the Parent Compounds in HDF Cells

**Figure 3:**
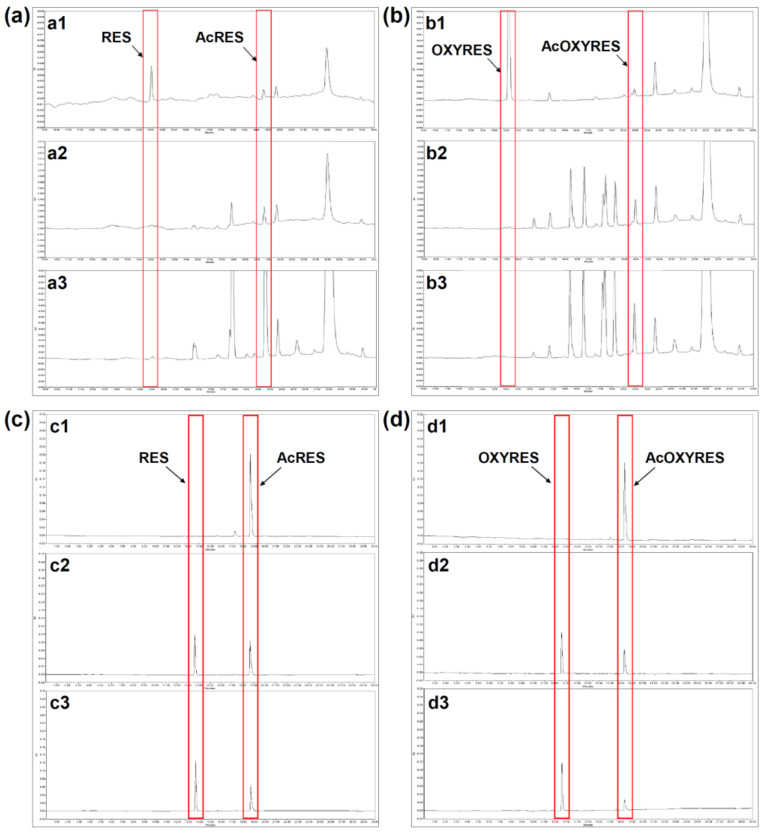

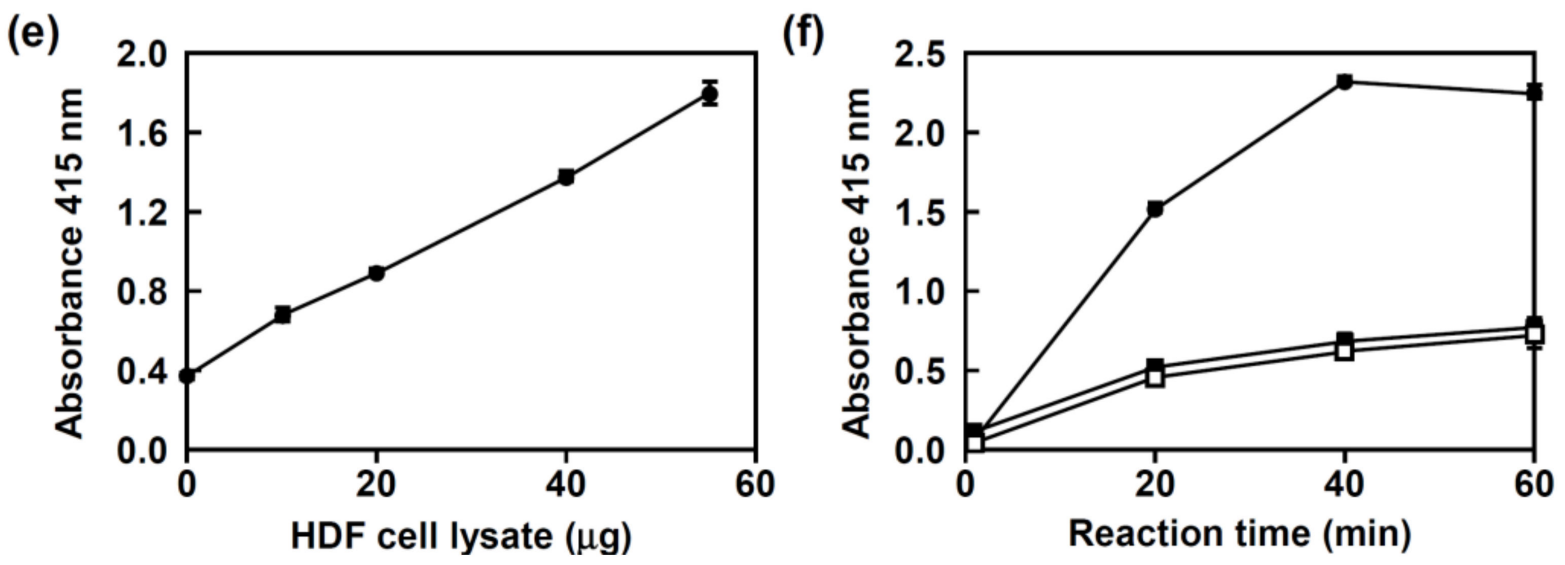
Deacetylation of AcRES and AcOXYRES by esterase from HDF cells. The HPLC chromatograms for AcRES incubated with (**a1**) HDF cell lysates, (**a2**) heat-treated HDF cell lysates, and (**a3**) only lysis buffer. The HPLC chromatograms for AcOXYRES incubated with (**b1**) HDF cell lysates, (**b2**) heat-treated HDF cell lysates, and (**b3**) only lysis buffer. Data correspond to AcRES and AcOXYRES, which are deacetylated to RES and OXYRES by the enzyme present in HDF cell lysates. The HPLC chromatograms for AcRES incubated (**c1**) without esterase, or with (**c2**) 2 U/mL of esterase, and (**c3**) 10 U/mL of esterase. The HPLC chromatograms for AcOXYRES incubated without (**d1**) esterase or with (**d2**) 2 U/mL of esterase and (**d3**) 10 U/mL of esterase. (**e**) Esterase activity of HDF cell lysate dependent on protein concentration. (**f**) Esterase activity of HDF cell lysates (filled circles), heat-treated HDF cell lysates (filled squares), and only lysis buffer (empty squares) dependent on reaction time.

Previous research confirmed the conversion of AcRES and AcOXYRES to their respective parent compounds by the cell lysate of B16F10 and suggested that intracellular esterase hydrolyzes the acetyl groups [19]. To confirm whether the antioxidant activities of AcRES and AcOXYRES, which were effective only in the cells and not in vitro, are due to their deacetylation to RES and OXYRES by hydrolysis of the acetyl group, HDF cell lysate was incubated with the acetylated compounds for 24 h. HPLC analysis data implied that the enzyme in HDF cells deacetylates AcRES and AcOXYRES to their respective parent compounds, RES and OXYRES. Standard RES, OXYRES, AcRES, and AcOXYRES had retention times of 14.52, 19.30, 13.59, and 18.98 min, respectively. The HPLC chromatogram of AcRES incubated with HDF cell lysate exhibited a strong peak at 14.52 min, corresponding to the retention time of RES (Figure 3(a1)). However, the peak for AcRES was substantially weaker than that for RES, indicating that most of the AcRES was deacetylated by the enzyme in the HDF cell lysate (Figure 3(a1)). In addition, the peak for RES was not present in the chromatogram of AcRES incubated with the heat-treated cell lysate or only lysis buffer, and the AcRES peak remained strong (Figure 3(a2,a3)). The chromatogram of AcOXYRES incubated with HDF cell lysate also exhibited a strong peak for its parent compound OXYRES and a relatively weaker peak for AcOXYRES, consistent with the results for AcRES incubated with HDF cell lysate (Figure 3(b1)). The chromatogram of AcOXYRES incubated with either the heat-treated cell lysate or only lysis buffer showed a strong peak for AcOXYRES but did not exhibit any measurable peak for OXYRES (Figure 3(b2,b3)). The HPLC data supported that the enzyme in the HDF cell is responsible for the conversion (deacetylation) of AcRES and AcOXYRES to their respective parent compounds, RES and OXYRES.

To clarify the role of esterase in the deacetylation of AcRES and AcOXYRES by HDF cell lysate, porcine liver esterase was reacted with AcRES or AcOXYRES for 24 h, and the residual acetylated compounds and their converted (deacylated) compounds were detected. In the chromatograms, the peaks for AcRES and AcOXYRES decreased dependent on the esterase concentration, and concomitant peaks for their respective parent compounds, RES and OXYRES, were detected (Figure 3c,d). This deacetylation of AcRES and AcOXYRES by HDF cells was thus at least partially due to the intracellular esterase. Therefore, we further measured the esterase activity of HDF cell lysate using 4-nitrophenyl acetate, a substrate for esterase. Catalyzed hydrolysis of 4-nitrophenyl acetate by esterase releases 4-nitrophenyl, which can be spectrophotometrically detected. HDF cell lysate increased the deacetylation of 4-nitrophenyl acetate dependent on the protein (enzyme) concentration (Figure 3e), and this activity was abolished by inactivation of the enzyme with heat treatment (Figure 3f). The buffer for obtaining the cell lysate did not affect the deacetylation activity of the esterase (Figure S1). It follows that we demonstrated that HDF cell lysate has sufficient deacetylation activity to induce deacetylation of AcRES and AcOXYRES. We demonstrated that, taken together, AcRES and AcOXYRES are deacetylated by esterase in HDF cells into RES and OXYRES, respectively, resulting in a similar effect to the parent compounds in the cells.

### 3.3. RES, AcRES, OXYRES, and AcOXYRES Inhibit MMP-1 Enzyme Activity In Vitro and UVB-Induced MMP-1 Expression in HDF Cells

**Figure 4:**
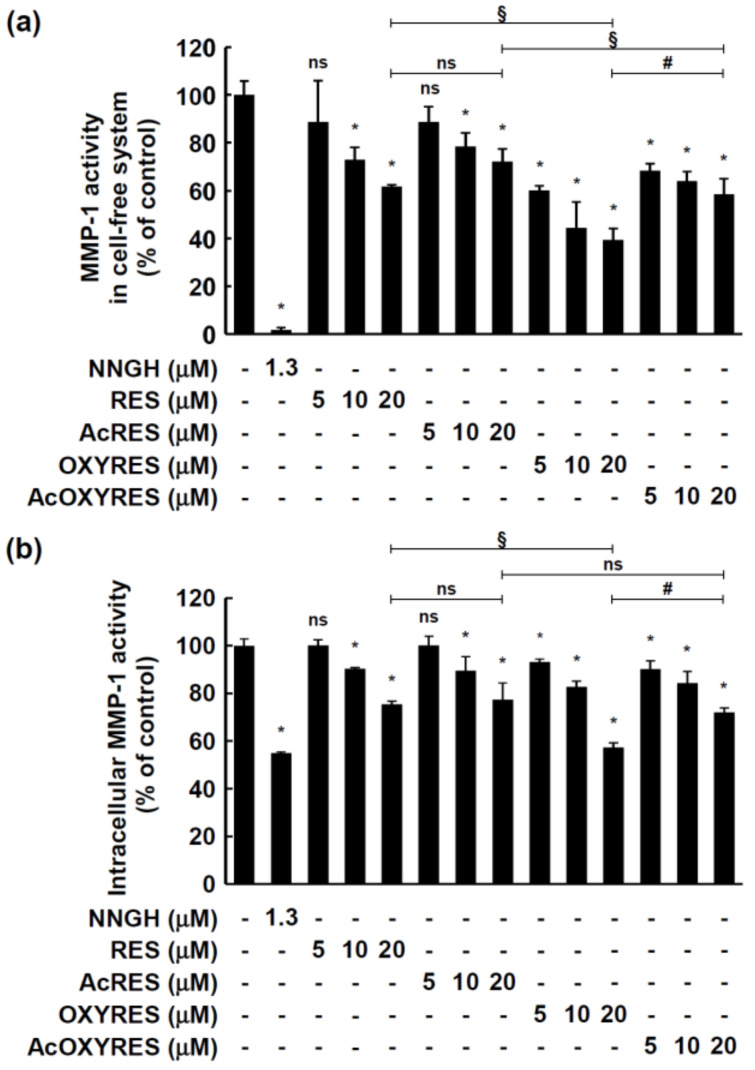
Effects of RES and its derivatives on matrix metalloprotease-1 (MMP-1) activity. (**a**) MMP-1 activity was determined as the activity of the recombinant human MMP-1 catalytic domain. (**b**) Intracellular MMP-1 activity in HDF cells was measured using the HDF cell lysates, as described in Materials and Methods. Data are mean ± SD of percentage relative to untreated control. *, Significant difference (*p* < 0.05) compared with untreated control; #, significant difference (*p* < 0.05) of parent vs. acetyl compounds; §, significant difference (*p* < 0.05) of (Ac)RES vs. (Ac)OXYRES; ns, not significant (*p* > 0.05).

The skin is largely divided into the epidermis and the dermis, of which the dermis occupies most of the skin structure, with a thickness 10 to 40 times that of the epidermis [29]. Collagen type I is the most abundant component of the ECM that comprises the dermis and is responsible for the strength and elasticity of the skin [30]. External stimuli such as UV irradiation stimulate the expression of MMP-1 in dermal fibroblasts to degrade collagen in the skin, and accumulation of such damage can induce wrinkle formation, a characteristic of extrinsic aging [31,32]. Therefore, MMP-1 is a major marker of skin aging [8,9,10] induced dependently or independently of ROS by UVB. Demonstration of the antioxidant activities implicated that AcRES and AcOXYRES could prevent skin aging. To more directly demonstrate the anti-aging effects of AcRES and AcOXYRES on the skin, we evaluated the impact of these compounds on the activity of MMP-1, which plays an important role in collagen degradation in the human skin dermis. RES and AcRES significantly inhibited the activity of MMP-1 in the cell-free system at concentrations above 10 μM, and OXYRES and AcOXYRES significantly inhibited the activity at concentrations above 5 μM (Figure 4a). RES, AcRES, OXYRES, and AcOXYRES at 20 μM reduced the activity of recombinant MMP-1 to 61.75, 72.34, 39.41, and 58.61%, respectively, and the inhibitory effect of OXYRES was significantly higher at this concentration than that of the other compounds. We used HDF cell lysate to compare the inhibitory activity of these compounds against the full-length MMP-1 protein secreted by HDF cells rather than the partially recombinant MMP-1 containing the catalytic domain. RES and OXYRES and their acetylated derivatives suppressed intracellular MMP-1 of HDF cells in a dose-dependent manner (Figure 4b). Interestingly, the inhibition activity of each compound on intracellular MMP-1 showed the same trend as that in the cell-free system.

**Figure 5:**
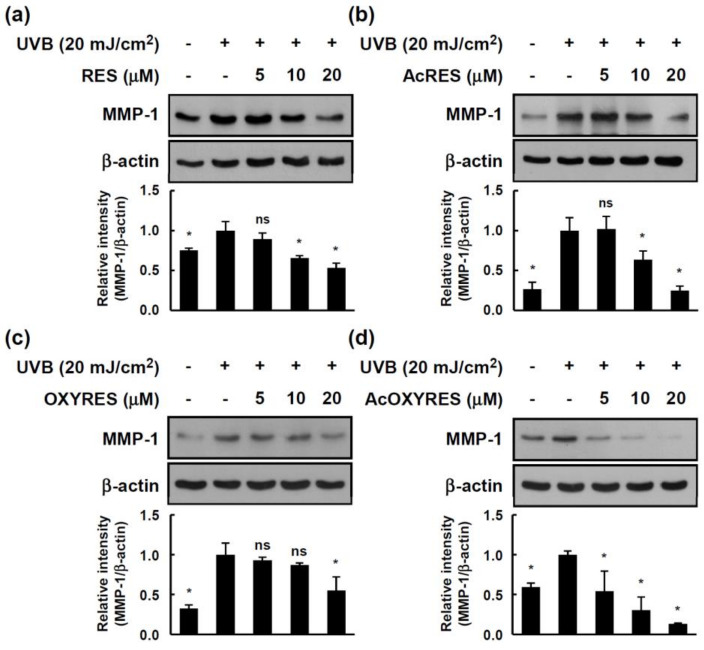
Effects of RES and its derivatives on UVB-induced MMP-1 expression in HDF cells. Starved HDF cells were treated with (**a**) RES, (**b**) AcRES, (**c**) OXYRES, or (**d**) AcOXYRES at the indicated concentration for 1 h before being exposed to UVB (20 mJ/cm^2^) for 24 h. MMP-1 and β-actin expression levels were measured by Western blot analysis using specific antibodies, as described in Materials and Methods. The band intensity of MMP-1/β-actin was represented as the mean ± SD of the fold change value relative to the UVB-treated group. Data represent three independent experiments that gave similar results. *, Significant difference (*p* < 0.05) compared with UVB-irradiated control.

MMP-1-mediated skin aging can be delayed not only by inhibition of enzymatic activity but also by suppression of the protein overexpression induced by specific stimuli [33]. Therefore, we evaluated whether pretreatment of HDF cells with RES, OXYRES, and their acetylated derivatives before UVB irradiation attenuated the MMP-1 expression to provide further evidence of their anti-wrinkle effects. RES, AcRES, OXYRES, and AcOXYRES were added to the HDF cell culture medium at concentrations of 0, 5, 10, or 20 μM, followed 1 h later by UVB irradiation (20 mJ/cm^2^). Twenty-four hours after UVB irradiation, HDF cells were harvested, and the expression of MMP-1 in the cell lysate was detected by Western blot analysis. RES and AcRES at 10 μM (Figure 5a,b) and OXYRES at 20 μM (Figure 5c) significantly inhibited UVB-induced MMP-1 expression. AcOXYRES, an acetylated derivative of OXYRES, abolished the UVB-induced MMP-1 expression at all concentrations (Figure 5d). RES has already been shown to inhibit MMP-2 and -9 activity and decrease the expression levels of these MMPs in various cells [34,35]. A recent study found that RES inhibited the gene expression of MMPs, including MMP-1, induced by interleukin (IL)-1β in articular chondrocytes [36]. In other work, RES and RES-enriched rice extracts negatively regulated the UVB-induced increase in MMP-1 in HDF cells [37]. The present study affirmed that RES attenuates the expression of UVB-induced MMP-1 in HDF cells, supporting the role of RES as a suppressor of MMP-1. It also confirmed that OXYRES suppressed UVB-induced MMP-1 expression in HDF cells markedly and that AcRES and AcOXYRES have this effect. These results showed that the acetylated derivatives of RES and OXYRES can inhibit the activity and expression of MMP-1 similar to or better than their respective parent compounds, suggesting that AcRES and AcOXYRES could be used to supersede their unstable parent compounds, resulting in suppression of MMP-1-mediated dermal damage inflicted by UVB.

### 3.4. RES, AcRES, OXYRES, and AcOXYRES Are Involved in UVB-Induced MMP-1 Expression by Modulating Activation of MAPK and Akt/mTOR Signaling Pathway

**Figure 6:**
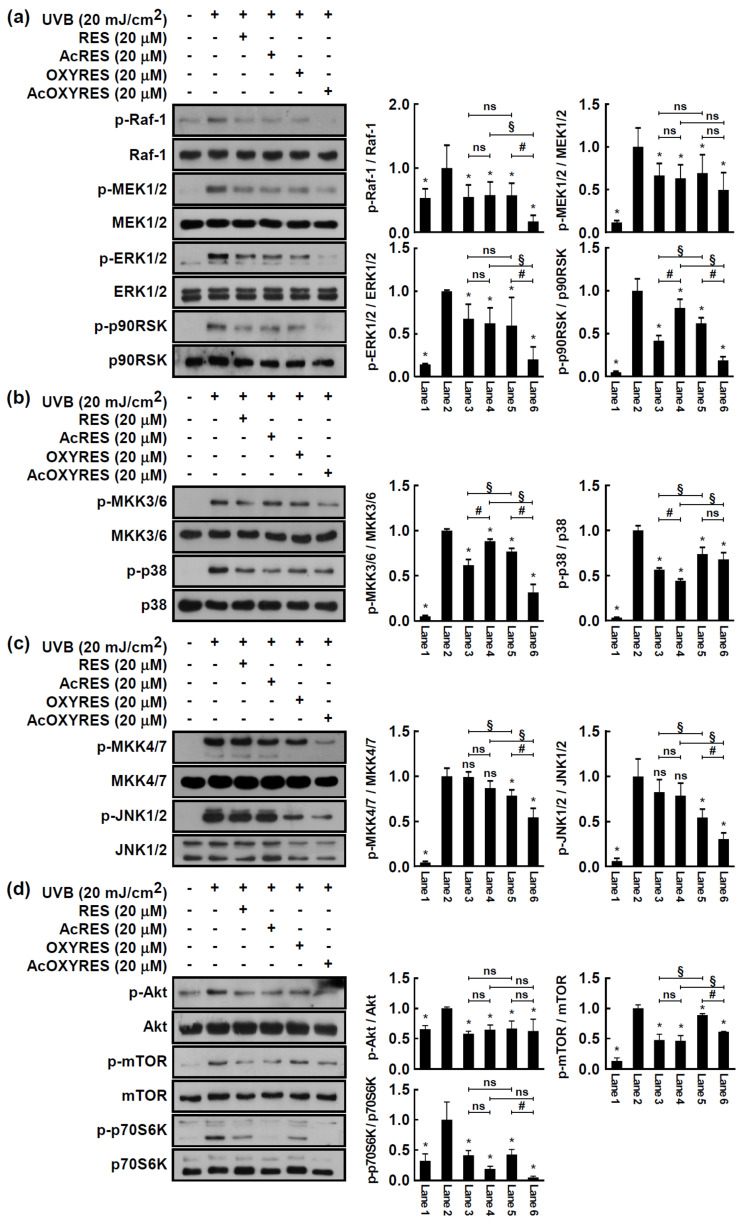
Effects of RES and its derivatives on UVB-induced (**a**–**c**) MAPK and (**d**) Akt/mTOR signaling pathways in HDF cells. HDF cells were starved for 12 h in serum-free DMEM and treated with the compounds. After 1 h, the cells were irradiated with UVB (20 mJ/cm^2^) and harvested 30 min later. The levels of phosphorylated or total forms of kinase proteins were determined by Western blot analysis using specific antibodies, as described in Materials and Methods. The band intensity of phospho-/total kinase is represented as mean ± SD of the fold change relative to the UVB-treated group. Data represent three independent experiments that gave similar results. *, Significant difference (*p* < 0.05) compared with UVB-irradiated control; #, significant difference (*p* < 0.05) of parent vs. acetyl compounds; §, significant difference (*p* < 0.05) of (Ac)RES vs. (Ac)OXYRES; ns, not significant (*p* > 0.05).

We observed a reduction in the UVB-induced MMP-1 protein levels by AcRES and AcOXYRES, which suggested that AcRES and AcOXYRES could inhibit the induction of MMP-1 protein expression. However, these results cannot exclude the possible degradation of the already expressed MMP-1 protein. Therefore, we performed further cell assays and Western blot analysis to confirm that RES, AcRES, OXYRES, and AcOXYRES also regulate the cellular signaling system responsible for upregulating the protein expression of MMP-1 induced by UVB. Previous studies indicated the involvement of the MAPK signaling pathway in UVB-induced MMP-1 expression [11]. Therefore, identifying the effects of RES, AcRES, OXYRES, and AcOXYRES on UVB-induced MAPK phosphorylation in HDF cells is important for understanding the mechanisms underlying their regulation of MMP-1 expression. All four compounds significantly downregulated the activation of the UVB-induced ERK signaling pathway consisting of sequential phosphorylation of Raf-1, MEK1/2, ERK1/2, p90RSK, and p38 signaling pathways involving sequential phosphorylation of MKK3/6 and p38 in HDF cells (Figure 6a,b). Conversely, activation of the MKK4/7–JNK1/2 signaling pathway by UVB irradiation was inhibited by OXYRES and AcOXYRES in HDF cells but not by RES and AcRES (Figure 6c). According to Shin et al., activation of the Akt/mTOR signaling pathway is also involved in UVB-induced MMP-1 expression in HDF cells [38]. Therefore, we confirmed whether RES, AcRES, OXYRES, and AcOXYRES could regulate the Akt/mTOR signaling pathway. All four compounds suppressed the pathway by significantly downregulating UVB-induced phosphorylation of Akt, mTOR, and p70S6K in HDF cells (Figure 6d).

Our molecular mechanism study of the inhibition of MMP-1 expression by RES, OXYRES, and their acetylated derivatives showed that RES and AcRES partially abolished the activity of the UVB-activated ERK, p38, and Akt/mTOR signaling pathways, but not the JNK1/2 signaling pathway. In contrast, OXYRES and AcOXYRES dramatically inhibited the MAPKs and Akt/mTOR signaling pathways in UVB-irradiated HDF cells. Generation of UV-induced ROS as a secondary messenger activates the MAPK family to stimulate the AP-1 transcription factors, c-Jun and c-Fos, essential in the regulation of MMP-1 gene transcription in the skin [5,39]. Moreover, UVB is reported to enhance the activity of p70S6K, a downstream target of Akt/mTOR, resulting in the regulation of MMP-1 protein levels in HDF cells [40]. These ROS actions can lead to an increased MMP-1 expression in keratinocytes and dermal fibroblasts [41,42]. Some studies have shown that UVB stimulates MMP-1 expression directly, mainly in HDF cells, but other studies have suggested an indirect mechanism. ROS production by UV irradiation can increase cytokines, such as IL-6 and IL-1β in dermal fibroblasts, and these also mediate the enhancement of MMP-1 expression. Furthermore, one study found that UVB-irradiated keratinocyte culture medium, which has increased levels of IL-6 and IL-1β, indirectly increases the expression of MMP-1 in HDF cells [43]. These findings highlight the role of ROS in skin aging mediated by MMP-1. Based on the accumulated evidence for MMP-1 gene transcription by UVB in human skin, the attenuation of MMP-1 expression by RES, OXYRES, and their acetylated derivatives might be mediated via MAPKs and Akt/mTOR pathways in HDF cells. Interestingly, AcOXYRES was more advantageous than OXYRES for inhibiting phosphorylation of most of the investigated kinases. Instead, RES showed no such trend. Moreover, between the acetylated compounds, unlike their respective parent compounds, the inhibitory effect of AcOXYRES on UVB-induced kinase activity was generally superior to that of AcRES. This result challenged our hypothesis that acetylated compounds would be converted to RES and OXYRES, acting the same as the parent compounds. However, on the one hand, we expected that the efficacy of OXYRES, which has a greater reactivity, decreased because of its lower stability than the parent compound. On the other hand, when the stability was improved by acetylation, it would act on HDF more stably and thus be able to exhibit results different from that of the parent compound. As a result, the ability of AcOXYRES to inhibit the phosphorylation activities of kinases, which are involved in the UVB-induced MMP-1 expression, was the strongest with AcOXYRES treatment, corroborating the effects of the studied compounds on the regulation of MMP-1 expression. Our results suggest that intracellular conversion to the parent compounds by treatment with acetylated forms may be more advantageous for maintaining the stability of the compounds than treatment with the parental forms. Although the observations do not expound on the differential regulation of MAPK signaling by RES and OXYRES, with only one hydroxyl group difference, we expect that OXYRES, with a relatively higher antioxidant activity, will be more advantageous for MAPK inhibition because UV-induced ROS activated MAPK signaling. Furthermore, the intensity of the interaction with a target protein may vary due to the difference in the number of hydroxyl groups between RES and OXYRES, which may change the inhibitory activity of the enzyme. A recent study revealed that the higher tyrosinase inhibition by OXYRES compared to RES could be attributed to structural differences in binding capacity and hydrogen bonding amount [44]. This suggests that the activity of OXYRES (AcOXYRES), which inhibited more kinases more strongly in our results, could be due to one more attached hydroxyl residue.

### 3.5. RES, OXYRES, and Their Acetylated Derivatives Suppress Collagen Degradation in HDF Cells

**Figure 7:**
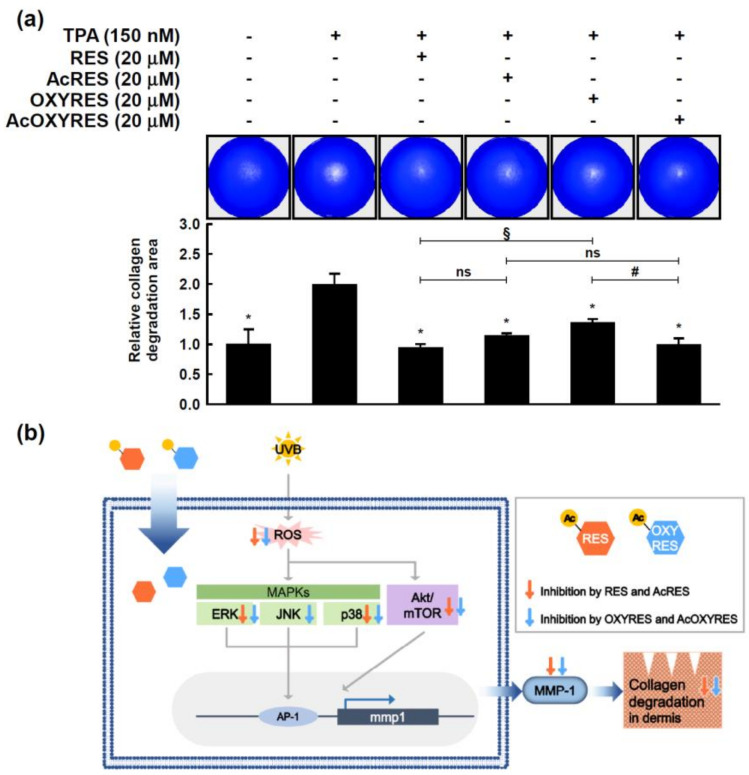
Effects of RES and its derivatives on UVB-induced collagen degradation. (**a**) After HDF cell attachment, serum-free DMEM with 150 nM 12-*O*-tetradecanoylphorbol-13-acetate (TPA) and 20 μM of samples was added. After 5 days, the HDF cells were removed, and the collagen cleavage was visualized by Coomassie blue staining. Collagen degradation was quantified using ImageJ software. Data represent mean ± SD of three independent experiments. *, Significant difference (*p* < 0.05) compared with UVB-irradiated control; #, significant difference (*p* < 0.05) of parent vs. acetyl compounds; §, significant difference (*p* < 0.05) of (Ac)RES vs. (Ac)OXYRES; ns, not significant (*p* > 0.05). (**b**) Schematic diagram of the proposed anti-skin aging mechanism of acetyl derivatives of RES and OXYRES. Acetylated RES and OXYRES lose their acetyl group and exert an inhibitory effect on ROS generation in HDF cells. In addition, RES, OXYRES, and their acetylated derivatives can suppress UVB-induced MMP-1 expression via inhibition of ERK, p38, Akt/mTOR and/or JNK signaling pathways. Furthermore, this mechanism can lead to inhibition of collagen degradation in HDF cells.

Type I collagen is found in all dermal layers [45]. Considering that MMP-1 initiates the degradation of collagen type I [37], the ability of AcRES, AcOXYRES, and their parent compounds to inhibit collagen degradation was evaluated. Collagen gels were constructed, embedded with cultured HDF cells, then treated with TPA to induce collagen degradation. On day 5, HDF cells treated with 150 nM TPA had cleaved the collagen underneath the cells seeded in the center of the collagen gel compared with the TPA-untreated groups (Figure 7a). When the HDF cells were treated with 20 μM of each compound (RES, AcRES, OXYRES, and AcOXYRES), the collagen degradation was inhibited relative to the TPA-treated groups. These results provided evidence for the availability of AcRES and AcOXYRES as anti-skin aging agents, showing that these compounds can successfully offset the degradation of type I collagen.

## 4. Conclusions

The present study attempted to elucidate the effects and molecular mechanisms of RES, OXYRES, and their acetylated derivatives (AcRES and AcOXYRES) on UVB-induced skin aging to verify the availability of AcRES and AcOXYRES as anti-aging cosmeceuticals for the skin. We found that AcRES and AcOXYRES were deacetylated to their respective parent compounds in HDF cells, in turn inhibiting ROS production and UVB-induced MMP-1 expression. These effects were similar to or better than those of the parent compounds. Furthermore, our results suggested that AcRES inhibits ERK, p38, and Akt/mTOR signaling, and AcOXYRES additionally inhibits JNK signaling, resulting in downregulation of the UVB-induced MMP-1 expression (Figure 7b). However, the process of converting AcRES and AcOXYRES to their parent compounds by HDF cells remains unknown, and further research is needed to investigate whether the proposed anti-aging effects on the skin in the cell culture model can be reproduced in vivo. Nevertheless, this work shows that the acetyl derivatives of RES and OXYRES restore UVB-compromised collagen, suggesting the possibility of AcRES and AcOXYRES as stable and effective anti-aging cosmeceuticals for the skin.

## Data Availability

Data is contained within the article and Supplementary Materials.

## Supplementary Materials

The following are available online at https://www.mdpi.com/article/10.3390/antiox10081252/s1, Table S1: Cytotoxic concentration 50 (CC50) of RES, OXYRES, and their acetyl derivatives. Figure S1: Enzymatic activity of esterase in cell lysis buffer.
