# Supplementary material for: Acetylated Resveratrol and Oxyresveratrol Suppress UVB-Induced MMP-1 Expression in Human Dermal Fibroblasts
Source: Antioxidants (Basel). 2021 Aug 5;10(8):1252. doi: 10.3390/antiox10081252 (PMC8389240; doi:10.3390/antiox10081252)
Supplement: Supplementary file 1 [file antioxidants-10-01252-s001.zip › antioxidants-1317559-supplementary.pdf]

antioxidants

Suppplementary Materials: Acetylated Resveratrol and Oxyresveratrol Suppress UVB-Induced MMP-1 Expression in Human Dermal Fibroblasts

Jae-Eun Lee [1,†], Jijeong Oh [1,†], Daeun Song [1], Mijeong Lee [1], Dongyup Hahn [1,2], Yong Chool Boo [3] and Nam Joo Kang [1,2,*]

[1] School of Food Science and Biotechnology, Kyungpook National University, Daegu 41566, Korea; lju1033@naver.com (J.-E.L.); ojjeong0113@hanmail.net (J.O.); sde940902@naver.com (D.S.); lmj7083@hanmail.net (M.L.); dohahn@knu.ac.kr (D.H.)
[2] Department of Integrative Biology, Kyungpook National University, Daegu 41566, Korea
[3] Department of Molecular Medicine, Cell and Matrix Research Institute, BK21 Plus KNU Biomedical Convergence Program, School of Medicine, Kyungpook National University, Daegu 41944, Korea; ycboo@knu.ac.kr
* Correspondence: njkang@knu.ac.kr; Tel.: +82-53-950-5753
† These authors contributed equally to this work.

Table S1. Cytotoxic concentration 50 (CC50) of RES, OXYRES, and their acetyl derivatives.

Compounds	CC50 [1]
RES	300.5 ± 55.6
AcRES	183.0 ± 22.4
OXYRES	381.0 ± 37.4
AcOXYRES	182.6 ± 59.8

[1] CC50 is the concentration that reduced the cell viability to 50% and presents as the mean ± SD (n=3).

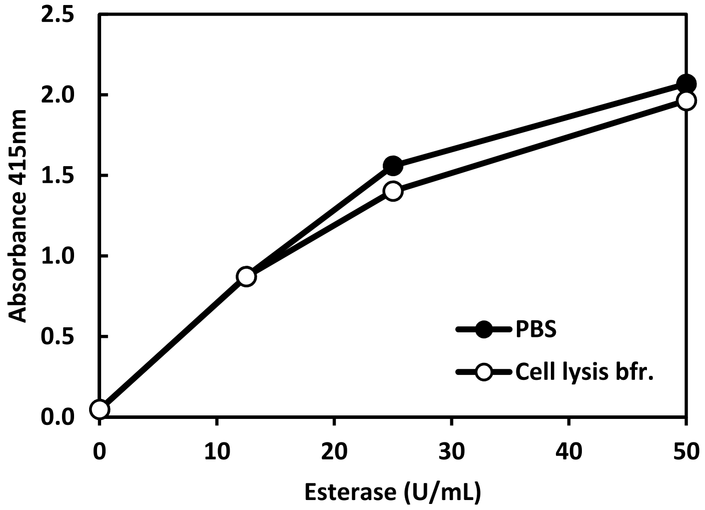

Figure S1. Enzymatic activity of esterase in cell lysis buffer.
